# Design of an Air Pollution Monitoring Campaign in Beijing for Application to Cohort Health Studies

**DOI:** 10.3390/ijerph14121580

**Published:** 2017-12-15

**Authors:** Sverre Vedal, Bin Han, Jia Xu, Adam Szpiro, Zhipeng Bai

**Affiliations:** 1 Department of Environmental and Occupational Health Sciences, University of Washington School of Public Health, Seattle, WA 98105, USA; caroljx@uw.edu; 2 State Key Laboratory of Environmental Criteria and Risk Assessment, Chinese Research Academy of Environmental Sciences, Beijing 100112, China; hanbin82@uw.edu (B.H.); baizp@craes.org.cn (Z.B.); 3 Department of Biostatistics, University of Washington School of Public Health, Seattle, WA 98195, USA; aszpiro@uw.edu

**Keywords:** air pollution, mobile monitoring, exposure estimation, cohort study

## Abstract

No cohort studies in China on the health effects of long-term air pollution exposure have employed exposure estimates at the fine spatial scales desirable for cohort studies with individual-level health outcome data. Here we assess an array of modern air pollution exposure estimation approaches for assigning within-city exposure estimates in Beijing for individual pollutants and pollutant sources to individual members of a cohort. Issues considered in selecting specific monitoring data or new monitoring campaigns include: needed spatial resolution, exposure measurement error and its impact on health effect estimates, spatial alignment and compatibility with the cohort, and feasibility and expense. Sources of existing data largely include administrative monitoring data, predictions from air dispersion or chemical transport models and remote sensing (specifically satellite) data. New air monitoring campaigns include additional fixed site monitoring, snapshot monitoring, passive badge or micro-sensor saturation monitoring and mobile monitoring, as well as combinations of these. Each of these has relative advantages and disadvantages. It is concluded that a campaign in Beijing that at least includes a mobile monitoring component, when coupled with currently available spatio-temporal modeling methods, should be strongly considered. Such a campaign is economical and capable of providing the desired fine-scale spatial resolution for pollutants and sources.

## 1. Introduction

Estimating exposure to air pollution in members of an epidemiological cohort study is central to assessing associations between air pollution exposure and adverse health effects in these influential studies. It is notable that such a critical aspect of this research enterprise is also very challenging. Not only is estimation of air pollution exposure technically and logistically challenging [1,2], investigators are faced with uncertainty regarding the impacts of exposure measurement error on study health effect estimates. On the other hand, today we have access to an array of tools for estimating exposure that, while making the task more complex, undoubtedly enhance our ability to make more valid exposure estimates.

Here we approach the task of attempting to estimate exposure to specific air pollutants and air pollution sources for study cohort participants in Beijing, China. While there is a focus on Beijing, and Beijing is used as an example, the considerations and options are generally applicable to any air pollution cohort study anywhere, especially when within-urban exposure contrasts are of interest. The focus will be on cohort studies in which the primary exposure contrasts are spatial, long-term contrasts rather than temporal, such as in time series studies where interest is largely in effects of short-term (hours to days) exposure contrasts. Further, the focus will also be on within-city contrasts in air pollution exposure, in this case for Beijing. While studies involving multiple cities have advantages, including the typically large variability in pollution concentrations across city and a large sample size of study subjects, there is persistent concern over residual confounding of the health effect estimates due to unmeasured or uncontrolled differences between cities [3,4]. We consider the relative strengths and weaknesses of an array of tools and methods that we might use for this purpose, and assess their applicability specifically to Beijing. It is not the intent here to review these in depth, but rather to highlight the options to be considered. It is hoped that by going through this process we will be able to determine, or at least provide some guidance about, a preferred approach or approaches that are applicable in the current context.

## 2. Air Monitoring Considerations

Before considering the array of available options for estimating exposure to air pollution in Beijing, it will be valuable to outline the considerations that are relevant to making decisions regarding their relative merits. It is also not the intent here to review these in depth, but to call attention to issues that should ideally enter into the thought process of planning a campaign to predict exposures for a cohort study in Beijing.

### 2.1. Geographic Scale

The geographic scale at which health outcomes are available partly determines the scale at which exposure needs to be estimated. For example, there is no point in attempting to estimate exposure at the individual subject level if the outcome, mortality rate, for example, is only available at an administrative spatial level in Beijing, such as a city district. In that case, estimating exposure at approximately the same spatial scale as the outcome would be reasonable. Also, if interest is only in estimating health effects associated with a particular air pollutant whose concentrations vary only over relatively large spatial scales, as exemplified by various secondary pollutants such as sulfate, then methods that estimate exposures at very fine spatial scales are probably not required. Alternatively, if health outcome data are available at the level of the individual subject in a cohort, and interest is also in pollutants whose concentrations vary over fine spatial scales, then methods that estimate pollutant concentrations at fine scales are also desirable, for reasons that will be itemized below.

### 2.2. Measurement Error

The impact of exposure measurement error on estimates of health effects is a complex issue. Impacts range from that of simply reducing the precision of health effect estimates to that of producing bias in the effect estimates, with varying combinations of the two [5]. Traditionally, measurement errors have been classified into classical measurement error, which largely produces a downward bias in effect estimates, and Berkson error, which largely reduces the precision of effect estimates. This classification has recently been extended to account for impacts of measurement error resulting from spatial modeling of exposure with introduction of the concepts of classical-like and Berkson-like forms of exposure measurement error [6,7]. Attempting to minimize these errors has implications for the monitoring design and the model used to estimate exposure based on the monitoring data.

### 2.3. Spatial Misalignment and Incompatibility

Spatial misalignment refers to the fact that air pollutant concentrations are observed at monitor locations that are distinct from locations of the population cohort. Almost all cohort studies are affected by spatial misalignment, which is what necessitates predicting exposures at subject locations. Some cohort studies are also affected by spatial incompatibility, which refers to systematic differences in the distributions of locations of monitor sites in a monitoring network relative to locations of members of the population cohort. Spatial incompatibility can result in biased health effect estimates [7,8]. While there is some evidence that land use regression (LUR) models can be transferable to regions not included in the monitoring area [9], there is little guarantee that this will be the case in other, or even most, settings. It may also be possible to minimize the impact of spatial incompatibility by reweighting individual monitoring data, but methods to do this in practice have not yet been validated. Therefore, measures to minimize spatial incompatibility are best taken when designing a pollution monitoring network for a specific cohort.

### 2.4. Existing vs. Study-Specific Monitoring

A fundamental decision to be made is whether existing air pollution data from a monitoring network, usually an administrative or regulatory network, can be used, or whether new monitoring data will be required. This decision is made more complicated when considering that existing monitoring data can be supplemented by either satellite date or predictions from air quality models, or both, both of which are types of “existing” data. Such supplementation can be used to try to improve a model in areas for which monitor coverage is poor, which is often the case. The decision as to whether new monitoring data are needed is also influenced by several of the issues brought up here, such as scale of the predictions needed, degree of measurement error and spatial compatibility.

### 2.5. Concentration vs. Exposure

It is axiomatic that ambient concentrations do not equate with exposures of individuals. With considerable effort an attempt can be made to better estimate exposure to ambient pollutants by: (1) making indoor and other micro-environmental or personal concentration measurements, at least on a sample of cohort members; (2) obtain data on individual house characteristics; (3) build a model to estimate indoor concentrations based on outdoor concentration and house characteristics [10]; (4) gauge time-activity patterns on the entire cohort; and (5) estimate exposures for individual cohort members using time-weighted averages of predicted concentrations from the relevant micro-environments. Ultimately, while theoretically superior, given the uncertainties in these exposure estimates, it is not clear that such an attempt to better capture exposure in fact always results in either substantially better estimates of exposure to ambient pollutants or improved exposure health effect estimates.

### 2.6. Source vs. Pollutant

While the vast majority of the work done to date on air pollution exposure estimation and associated health effects has focused on single pollutants, especially the criteria air pollutants, there is good rationale for also focusing on specific sources of air pollution. Calls have been made for placing a source or mixtures orientation high on the air pollution research agenda [11]. From the perspective of air quality management, interventions or policies are aimed directly at pollution sources, or precursor sources, rather than specific pollutants, although the ultimate purpose is to reduce specific pollutant concentrations. Insight into the health effects associated with specific sources, and therefore specific pollutant mixtures, offers the potential of more effectively and efficiently reducing the most harmful types of air pollution by directly reducing emissions from the most harmful pollution sources. Taking a source-oriented approach to estimating air pollution exposure then raises the prospect of directly estimating health effects of specific pollution source types. Formal source apportionment approaches, such as a receptor-oriented approach like positive matrix factorization (PMF), UNMIX and chemical mass balance (CMB) can be used to generate source factors that can be then be employed in a spatio-temporal or other modeling format to obtain source-specific exposure predictions.

### 2.7. Exposure Time Windows

Different exposure data and exposure estimation methods have implications for the time period relative to a given health endpoint that exposure can be estimated. For example, a long historical record of satellite data might allow them to be used for estimating exposure at any number of time points and over any number of averaging periods, at the expense of estimating exposure with relatively less spatial resolution. The same can be said for air quality models. Exposure estimation approaches that rely heavily on directly measured air pollution concentrations over relatively short time periods, on the other hand, might not lend themselves to extrapolation to periods preceding the period of actual monitoring. While a long record of monitoring data might be available for some pollutants, such as PM_2.5_ or ozone, spatially dense monitoring data or data on multiple pollutants are unlikely to be available over an extended period of time. How such data are used in modeling exposure, and the assumptions required in extrapolating model estimates beyond the period of monitoring, will determine whether it is reasonable to use such data for many of the above exposure specifications. It is somewhat reassuring that LUR models, at least for some pollutants, can be generalized to other time periods [12]. The health endpoint in a cohort study that is being analyzed also influences to some extent the time period or periods for which exposure estimates are needed (see “Cohort study endpoints” below).

### 2.8. Evaluation of Predictions

Evaluation of air pollution exposure predictions is an important step in gauging the degree of exposure measurement error that may be present and, in turn, the likely impact of this error on the cohort air pollution health effect estimates. Since evaluation of predictions usually entails comparing predictions with monitored concentrations [13], which occur at specific points in space, exposure prediction approaches that allow point predictions lend themselves readily to application of formal methods of assessing prediction validity. Currently, these methods typically employ some form of cross-validation [14,15]. Gridded predictions can be assigned to point locations in a simple (e.g., assignment of closest monitor) or complicated (e.g., smoothing of gridded data) manner and then compared to monitoring data. For pollutants that typically are spatially less variable such as PM_2.5_ this may lead to good point predictions and hence favorable validation statistics, whereas for more spatially-varying pollutants such as CO or NO_2_ such approaches to validation may not be appropriate.

### 2.9. The Beijing Context

**Figure 1:**
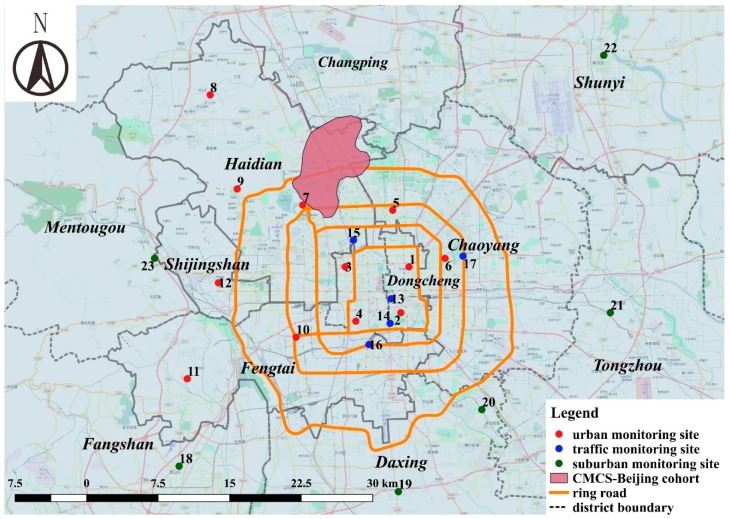
Map of Beijing showing the location of existing urban and suburban administrative air monitoring sites, and the location of the Beijing Chinese Multi-provincial Cohort Study cohort (CMCS-Beijing). Only 23 of the approximately 35 monitoring sites in the larger region are displayed. Monitor numbers correspond to those in Table 1. See Section 3.3 for a description of the CMCS–Beijing cohort. Map generated using QGIS (OSGeo, Beaverton, OR, USA) (www.qgis.org/).

Availability of the relevant monitoring and other data for Beijing will play a large part in determining which approaches to estimating population cohort exposures are feasible. The larger Beijing region has an extensive administrative air pollution monitoring network of approximately 35 monitoring sites that essentially continuously measure concentrations of most of the criteria air pollutants (Figure 1). Concentrations of PM_2.5_ and several gaseous pollutants in 2015 at 23 sites in central Beijing are shown in Table 1. PM_2.5_ concentrations are high and there seems to be relatively little spatial variation in concentration across the urban area, at least from these monitoring data. Predictions from LUR models based on such monitoring data would likely underestimate the true fine-scale variability in concentrations. Concentrations vary substantially across season, but the relative ranking of sites by concentration in each season is reasonably stable (data not shown). While public access to other than real time monitoring data, and historical data in particular, is limited, researchers have been able to access some historical data [16]. Remote-sensing satellite data are available [17]. The Global Burden of Disease enterprise has integrated several approaches to estimating exposures in China and Beijing, making use of pollutant monitoring data, satellite data and dispersion model predictions [17,18].

Much GIS data for developing LUR models in concert with monitoring data are readily available. For example, the Harvard Center for Geographic Analysis website (http://gis.harvard.edu/resources/data/china-gis-data) has links to many GIS data sites that are useful for LUR, including China Map (http://worldmap.harvard.edu/chinamap/), Open Street Map (OSM—http://download.geofabrik.de/asia/china.html) and Beijing City Lab (http://www.beijingcitylab.com/data-released-1/). There are many other available resources for obtaining geographic data for Beijing. Some of the relevant geographic variables and the type of variable (proximity (i.e., distance to) vs. buffer (amount within a defined radius)), as well as some of the potential information sources are listed in Table 2. This indicates that data availability is not a major issue in influencing the choice of exposure estimation method in Beijing.

## 3. Cohort-Related Considerations

### 3.1. Geographic Scale of Cohort Data

The more typical cohort study design employs health outcome and risk factor information at the level of the individual; that is, there are data on each individual in the cohort. Early influential air pollution cohort studies [19,20] employed individual-level health outcome and risk factor information that was a significant advance over purely ecologic studies with neither individual-level exposure or outcome information [21]. These studies did not, however, attempt to estimate exposure at the fine spatial scale of the individual subject.

Some air pollution epidemiology of long-term air pollution exposure effects, however, is carried out using datasets that do not include individual-level health outcome data. For example, in the United States, studies of long-term air pollution exposure effects have utilized Medicare data in which health outcome data, mortality rates, for example, were available only at the mailing code (zip code) level [22,23]. Point-level exposure predictions for such a cohort have no advantages over those with less spatial resolution.

Yet another type of observational study of long-term exposure effects does not even attempt to estimate exposures at any well-defined spatial scale, and utilizes outcome data that are specified over very large regions. For example, in the so-called natural experiments (or quasi-experimental studies) that exploit extra-ordinary exposure scenarios that counter concerns over traditional sources of spatial confounding of the exposure-response associations, there is typically no attempt to estimate exposures except very crudely over space. One recent example is the Huai River study in China that exploited China’s coal power policy of providing free coal for heating north of the Huai River, but not to the south, with resultant large regional differences in particulate matter concentrations [24].

Because we are interested here in employing spatial exposure contrasts within Beijing, and are assuming that we have health endpoints defined for cohort members for whom we have well-resolved residential locations, exposure estimation methods that predict exposures at finer spatial scales are preferred.

### 3.2. Health Endpoints in Cohort Studies

Cohort studies are typically time-to-event studies in which individual “survival” time, most often survival time to death, but also time to other endpoints, is compared relative to exposure. Less commonly, cohort data will have been collected on endpoints that were measured repeatedly over the course of follow-up, allowing for longitudinal analyses of exposure effects on change in the endpoint. For example, repeated measurements of lung function or imaging measures of atherosclerosis allow analyses of exposure-related differences in rate of decline or of progression to be carried out. Sometimes the timing of events is difficult to discern, or is identified with little certainty, in which case data from a cohort may be essentially analyzed in the same way as a cross-sectional study.

These several outcomes in the context of cohort studies have implications for the methods and data that are used for estimating exposure. Specifically, for time-to-event data, one might consider specifying exposure as cumulative exposure before the event, or as an average exposure over a defined period before the event, for example, one year or more before the event. Similarly, for longitudinal continuous data, cumulative exposure over the periods between each repeated measurement, or average exposure over a defined period before each measurement, could be reasonable options to consider. For a cross-sectional analysis, baseline exposure or exposure at the time of measurement might be the most reasonable exposure specification. These choices are influenced to some extent by the data that are available for estimating exposure. See “Section 2.7” above.

### 3.3. Beijing Cohorts

There are several currently active cohort studies in Beijing, including cardiovascular cohort studies, in which individual-level data are collected. As one example, the China Multi-Provincial Cohort Study (CMCS) is a cardiovascular cohort study involving 11 provinces in China, including parts in Beijing [25,26,27,28,29]. The CMCS in Beijing (CMCS-Beijing) is a survey of a general population in Beijing that was relatively healthy when enrolled in 1992 (*n* = 1982). A total of 1324 were examined again in 2002 [28]. Examination at that time included obtaining demographic information, including residential address, measurement of traditional cardiovascular risk factors, blood tests and carotid ultrasound measurements of atherosclerosis. In 2007, a total of 930 without cardiovascular disease underwent repeat measurement of risk factors and carotid ultrasound, which made possible analyses of longitudinal change in atherosclerosis. Table 3 shows baseline characteristics of this subset of the cohort, adapted from Qi et al. (2015). Figure 1 shows the spatial distribution of the CMCS-Beijing study subject residence locations in Beijing overlaid on the location of air monitoring sites. Only a few existing monitoring sites align spatially with the cohort locations, raising the prospect of spatial incompatibility and resulting biased health effect estimates if exposure predictions were based on this network of monitors.

Another example of an active cardiovascular cohort in Beijing with rich risk factor and endpoint data on over 20,000 adults is the Fangshan cohort that began data collection in 2008 [30]. There are also several active cohort studies in Beijing that focus on diseases other than cardiovascular disease. Most cohorts have baseline residential address and subsequent address data collected since the inception of the cohort.

## 4. Approaches for Estimating Exposure

Here we outline some of the more relevant approaches that could be taken to estimate within-city long-term air pollution exposures for a cohort study in Beijing. First, approaches that rely on directly measured air pollution concentrations will be presented. This will be followed by presentation of several approaches that do not rely on directly monitored data, and then by approaches that integrate, or fuse, two or more approaches. Finally, a discussion of the relative trade-offs, including advantages and disadvantages, of the several approaches will be presented. These are summarized below in Table 4.

### 4.1. Approaches Utilizing only Directly Measured Air Pollution Concentrations

#### 4.1.1. Using Administrative Monitoring Network Data

As noted above, air pollution cohort studies have typically only utilized existing administrative (i.e., collected by a government agency) monitoring data for estimating long-term exposure to ambient air pollution. Monitoring sites generally are relatively few in number, are not spatially dense, include measurements of one or relatively few pollutants and are sited away from pollution sources. Approaches to using such data to estimate exposures include: (i) network average; (ii) nearest monitor; (iii) inverse distance weighting; (iv) geostatistical smoothing, such as kriging; and (v) LUR. Obviously, using the network average, if the network extends over the entire urban area of interest, is of no use in estimating within-city exposure contrasts. Neither smoothing nor LUR are possible with a very small number of monitoring sites, and LUR is less useful if the siting of monitors does not nearly reflect the variety of air pollution exposures that are experienced by a given cohort, which is typically the case. LUR combined with smoothing can be used to enhance the performance of a LUR model by exploiting any spatial structure remaining in the regression residuals from the LUR [31,32]. This can be done either less formally as a two-step procedure [33] or in a unified framework such as LUR in a universal kriging framework [34].

#### 4.1.2. Using Study-Dedicated Monitoring Data

Some cohort studies, including the early Six Cities Study [19], utilized monitoring data from a monitoring network designed specifically for the study. This allowed for flexibility in monitor siting and the timing of monitoring, and had the potential to increase the number and characteristics of air pollutants measured. Very elaborate monitoring designs, such as the monitoring for the Multi-Ethnic Study of Atherosclerosis and Air Pollution Study [35,36], can include enhanced monitoring of pollutant components [37], enhanced monitoring of pollutant sources, such as roadways, and monitoring at cohort study participant locations [36]. These many types of monitoring data are often unbalanced, being collected at various times during a monitoring campaign, which complicates using them in generating exposure prediction models. The use of such unbalanced spatio-temporal data to generate prediction models has been facilitated by a recently-developed R package, the SpatioTemporal package [38]. Saturation monitoring using micro-sensors is another promising approach to collecting data from a large number of sites [39].

Mobile monitoring is yet another variation of a study-dedicated monitoring platform that has many potential advantages, as well as presenting some challenges [40,41,42]. Advantages of mobile monitoring include the relatively efficient measurement at a large number of sites and potentially of a large number of pollutants and pollutant characteristics. Disadvantages are that measurements: (1) are taken on the roadway, which are of questionable relevance for use in estimating residential concentrations; (2) are relatively few for each point in space; and (3) are affected by space-time confounding wherein measurements at different points in space are made at different times during the day when air pollution concentrations are changing; mobile measurements therefore reflect not only where they are made but also when they are made. The concerns that measurements are made on roadways and that there is relatively little data at each point can be alleviated by employing a mobile design whereby the monitoring vehicle is parked at specified residential locations for fixed periods of time rather than relying on measurements made only while on the move. Such a modification to the mobile monitoring design has been used successfully [43,44,45]. There are precedents for using a mobile monitoring platform in China, and in Beijing in particular [46,47].

### 4.2. Approaches Not Utilizing Measured Air Pollution Concentrations

These approaches have an important advantage over monitoring approaches in being able to estimate exposures at locations that are not typically monitored, such as rural locations, for example. This advantage comes at the expense of limited spatial resolution.

#### 4.2.1. Dispersion/Diffusion Models

These models use source emission characteristics together with meteorological data and/or terrain features of the source and receptor locations, and intervening locations, to estimate concentrations at receptor locations. Examples include AERMOD (an atmospheric dispersion model with several modules), CALINE (a California steady-state roadway line dispersion model), and CALPUFF (a California non-steady state puff dispersion model). Predictions can be resolved down to fine spatial scales such as 1 × 1 km grids [48].

#### 4.2.2. Chemical Transport Models

In addition to the input terms that are typically utilized in dispersion models, chemical transport models also incorporate atmospheric chemical (including photochemical) reactions of emitted pollutants in order to better estimate concentrations of pollutants undergoing chemical transformation or secondarily formed pollutants. Examples include the U.S. Environmental Protection Agency Community Multi-scale Air Quality modeling system [49] and the European Monitoring and Evaluation Programme—Meteorological Synthesizing Centre-West model [50]. (http://webdab.emep.int/Unified_Model_Results/). Spatial resolution of predictions has typically been no better than 10 × 10 km. However, selected predictions have recently been made at a 4 × 4 km grid [51,52].

#### 4.2.3. Satellite Remote Sensing Measurements

Use of remote sensing satellite platforms to measure ground-level pollutant concentrations has generated a great deal of enthusiasm in the air pollution and epidemiology communities [53]. Much has been learned about the strengths and limitations of such data [2,54] and about comparisons with other exposure prediction methods [55]. Advances in the technology and in the application of the data in exposure estimation are rapid. While spatial resolution was initially at a 10 × 10 km grid or larger, recent advances have allowed measurements to be made at a 1 × 1 km grid.

### 4.3. Approaches That Integrate Two or More Approaches

Because all approaches have relative strengths and weaknesses, there is rationale for combining or integrating two or more approaches to attempt to counter the weaknesses and exploit the strengths of individual approaches. For example, the relatively poor spatial resolution of non-monitoring approaches can be improved by supplementing them with approaches using LUR. There are several examples of how this integration has been done. Investigators at Georgia Institute of Technology have fused predictions from a chemical transport model with monitoring data [56], and in China, combined satellite data with monitoring data [57]. Investigators at Harvard University have combined satellite observations with monitoring data and LUR [58,59]. The Global Burden of Disease enterprise combined satellite data with monitoring data and predictions from a dispersion model [17]. At the University of Washington, LUR model predictions were supplemented by predictions from a diffusion model [60], and spatio-temporal models based on monitoring data incorporated predictions from a chemical transport model [52] or from satellite data [61]. Canadian investigators generated a model in which kriging based on monitoring data was combined with satellite observations [55]. Most of these integrated approaches are able to produce predictions at very fine spatial resolution, even at specific points in space.

## 5. Overall Assessment and Recommendations

For a cohort study in Beijing such as envisioned here, the following points are relevant in designing an optimal air pollution monitoring platform and exposure prediction method: (1) the platform employs at least some direct measurements of air pollution concentrations to allow point predictions; (2) more spatially dense monitoring is generally more desirable than less spatially dense monitoring; (3) monitoring should align spatially with the cohort of interest (exhibit spatial compatibility); (4) the approach can be used to estimate exposure to air pollution sources as well as exposure to individual air pollutants; (5) the approach is flexible enough to incorporate estimates of exposure from other approaches utilizing other direct pollutant monitoring data or non-monitoring data to attempt to improve predictions; (6) the exposure predictions generated are amenable to validation; and (7) the approach is realistic from the standpoint of cost and technical feasibility.

For the purpose of predicting air pollution exposures for a cohort, Beijing is fortunate in having an extensive administrative air monitoring network and a rich array of GIS data that can be used for LUR (see “Section 2.9 and Section 3.3”, above). However, depending on the spatial distribution in Beijing of the cohort used, for example, the distribution of the CMCS-Beijing cohort, even though there is a large monitoring network, spatial incompatibility could potentially be an important concern.

The traditional cohort study design, as noted above, is one in which the health endpoints and covariate data, such as risk factors for the endpoints, are available at the level of the individual study subject. However, even in a traditional cohort design, some covariate data, neighborhood measures of socio-economic status [62], or walkability, for example, may not be individual-level data, by their nature; these are instead assigned to all individuals within a given spatial boundary or at a given scale at which such data are reported or specified, such as a census tract, for example. There is a conceptual difference between covariate data that measure some characteristic of individuals that has meaning only at a group (spatial) level, such as neighborhood level socio-economic status (or walkability), and air pollution exposure estimates for most air pollutants that have meaning at the level of the individual even though they may not be estimable at that level; that is, air pollution concentrations can, in theory at least, differ between individuals included in a group defined by spatial boundaries, whereas group-level covariates cannot. A critical issue for a traditional cohort design is the preferred spatial resolution of estimated air pollution exposure. Often exposure estimates are only available at relatively low spatial resolution. In such instances, there is no alternative but to use these estimates, even though one might have preferred to use estimates that are resolved to the level of the individual study subject.

Determining which method(s) to use in estimating exposure in a cohort depends on many factors that often involve trade-offs, as summarized in Table 1. Of the approaches considered here, only those that allow for predictions at points in space, that is, those that include at least some direct monitoring of air pollutant concentrations, produce individual-level exposure estimates. In addition, while these estimates exhibit varying degrees of exposure measurement error, depending on how good the estimates are, they are amenable to improvements to lessen the measurement error. Estimates of exposure having less spatial resolution, no matter how good, cannot overcome the limitation that all subjects within a grid are assigned the same estimate of exposure. The degree of exposure measurement error introduced by grid-level estimates depends in part on the spatial scale of meaningful heterogeneity in concentration exhibited by a specific pollutant. In general, concentrations of secondary pollutants with a more regional spatial scale of heterogeneity, such as sulfate, would be expected to be predicted with less error than those emitted primarily from many point sources (so called area sources) within an airshed, such as carbon monoxide.

Of the approaches that generate predictions at points in space, there are good reasons for strongly considering a mobile monitoring platform. Mobile monitoring allows data to be efficiently obtained from many sites that can be spatially aligned with a specific cohort, and can be designed to efficiently measure concentrations of multiple pollutants and pollutant characteristics to allow estimates of source exposures over space to be made. Incorporation of fixed sites within the mobile monitoring design (parking for fixed periods of time, for example) addresses concerns about having a sufficient number of measurements at sites and having only roadway measurements. The issue of spatio-temporal confounding inherent in mobile measurements requires that considerable attention be paid to having sufficient data to perform some sort of time-adjustment of the measurements.

The approaches that combine (or fuse) some degree of monitoring data with other data or predictions are also capable of generating reasonably valid predictions at points in space, and so could also be considered. However, none of these integrated approaches lend themselves easily to generation of pollutant source predictions at the individual level.

## 6. Conclusions

In order to efficiently generate validated predictions of individual-level exposure to air pollutants and pollutant sources in Beijing, China, strong consideration should be given to employing a mobile monitoring campaign, coupled with advanced spatio-temporal modeling methods. Integrative approaches may also have merit for this purpose and could also be considered.

## Figures and Tables

**Table 1:** Annual PM_2.5_ and gaseous pollutant concentrations in 2015 for the individual administrative monitoring sites in central Beijing (see monitor locations in Figure 1).

Monitoring Site	PM_2.5_ (μg/m^3^)	SO_2_ (ppb)	NO_2_ (ppb)	Ozone (ppb)	CO (ppm)
1	81.5	5.3	24.8	26.3	1.1
2	77.4	4.2	25.5	28.4	1.0
3	78.7	4.9	27.1	26.8	1.0
4	80.8	5.0	26.0	27.9	1.1
5	78.0	5.0	30.0	30.2	1.1
6	80.5	5.6	28.8	28.9	1.1
7	77.1	5.2	28.7	24.6	1.1
8	78.7	4.8	23.6	17.3	1.2
9	68.5	4.1	18.1	33.6	0.8
10	87.1	5.6	29.8	23.1	1.2
11	80.4	4.4	20.0	29.6	1.0
12	80.1	4.7	24.5	28.6	1.1
13	86.0	5.4	32.1	22.1	1.1
14	85.9	6.3	34.9	21.7	1.2
15	82.7	6.1	36.7	20.4	1.1
16	94.3	7.2	47.6	13.0	1.3
17	86.9	5.7	34.2	21.9	1.2
18	89.7	5.6	27.5	24.4	1.2
19	91.9	6.3	27.1	28.2	1.2
20	90.6	5.9	26.6	28.9	1.1
21	91.1	7.0	27.9	27.0	1.1
22	76.0	3.7	21.3	24.4	0.9
23	68.9	3.4	19.1	26.9	0.9

**Table 2:** Selected relevant geographic variables for Beijing and selected potential sources of information.

Description	Source(s) (Agency/Website)
Proximity measures:	
nearest major road	Beijing Institute of Surveying and Mapping; Open Street Map (OSM—http://download.geofabrik.de/asia/china.html)
road intersection	Beijing Institute of Surveying and Mapping; Open Street Map (see above)
railway and railyard	Beijing Institute of Surveying and Mapping; Open Street Map (see above)
airport	Beijing Institute of Surveying and Mapping; Open Street Map (OSM—see above)
Buffer measures:	
major road length	Beijing Institute of Surveying and Mapping; Open Street Map (OSM—see above)
land-use category	Beijing Planning and Land Resource Management Committee
vegetation index	NASA (http://neo.sci.gsfc.nasa.gov/view.php?datasetId=MOD13A2_M_NDVI)
population density	Chinese Population GIS (http://cpgis.fudan.edu.cn/cpgis/default.asp/)
pollution sources	Beijing Municipal Research Institute of Environmental Protection emission inventories
Others:	
altitude	Beijing Institute of Surveying and Mapping; Google Earth

**Table 3:** Baseline (2002) characteristics of CMCS-Beijing cohort participants followed up in 2007.

Characteristic	Total (*n* = 930)	Men (*n* = 418)	Women (*n* = 512)
Age (years)	59.6 ± 7.8	61.1 ± 7.4	58.3 ± 8.0
Systolic blood pressure, mmHg	129.6 ± 18.2	132.3 ± 17.6	127.5 ± 18.5
Diastolic blood pressure, mmHg	80.8 ± 10.1	83.2 ± 10.2	78.8 ± 9.5
Body mass index	25.0 ± 3.3	25.1 ± 2.9	24.9 ± 3.5
Fasting blood glucose	4.90 ± 0.99	4.85 ± 1.04	4.95 ± 0.93
Current smoking	89 (9.6)	88 (21.1)	1 (0.2)
Hypertension	445 (47.8)	227 (54.3)	218 (42.6)
Diabetes	99 (10.6)	50 (12.0)	49 (9.6)
Carotid plaque	181 (19.5)	100 (23.9)	81 (15.8)
Maximal IMT, mm	0.90 (0.70–1.20)	1.00 (0.80–1.40)	0.90 (0.70–1.10)
Total cholesterol, mmol/L	5.17 ± 1.02	5.39 ± 0.96	5.72 ± 1.04
HDL cholesterol, mmol/L	1.40 ± 0.31	1.29 ± 0.26	1.78 ± 0.32

Adapted from Qi et al. (2015). Values are mean ± SD, median (interquartile range), or *n* (%). CMCS = Chinese Multi-provincial Cohort Study; IMT = carotid intima-media thickness; HDL = high-density lipoprotein.

**Table 4:** Comparative advantages and disadvantages of potential sources of air pollution data for exposure prediction in cohort studies.

Approach	Advantages	Disadvantages
includes pollutant monitoring:		
fixed, existing administrative	readily available; inexpensive	generally few sites; limited number of pollutants; may exhibit spatial misalignment/ incompatibility with study cohort
fixed, study specific	potential to be well-aligned with cohort; flexibility in pollutants to be monitored	expensive
mobile	spatially dense; relatively inexpensive; data on multiple pollutants collected efficiently	spatio-temporal confounding; roadway monitoring
saturation micro-sensor	spatially dense; multiple pollutants	relatively few pollutants; relatively long measurement time scale
includes no direct pollutant monitoring:		
dispersion models	good temporal resolution; estimates made over a long time span	relatively large spatial scale
chemical transport models	good temporal resolution; estimates made over a long time span	relatively large spatial scale
satellite	good temporal resolution; relatively long historical record	relatively large spatial scale; interference by cloud cover

## References

[1] McGuinn L.A., Ward-Caviness C., Neas L.M., Schneider A., Di Q., Chudnovsky A., Schwartz J., Koutrakis P., Russell A.G., Garcia V. (2017). Fine particulate matter and cardiovascular disease: Comparison of assessment methods for long-term exposure. Environ. Res..

[2] Paciorek C.J., Liu Y., HEI Health Review Committee (2012). Assessment and Statistical Modeling of the Relationship between Remotely Sensed Aerosol Optical Depth and PM_2.5_ in the Eastern United States.

[3] Sun M., Kaufman J.D., Kim S.Y., Larson T.V., Gould T.R., Polak J.F., Budoff M.J., Diez Roux A.V., Vedal S. (2013). Particulate matter components and subclinical atherosclerosis: Common approaches to estimating exposure in a Multi-Ethnic Study of Atherosclerosis cross-sectional study. Environ. Health.

[4] Kim S.Y., Sheppard L., Kaufman J.D., Bergen S., Szpiro A.A., Larson T.V., Adar S.D., Diez Roux A.V., Polak J.F., Vedal S. (2014). Individual-level concentrations of fine particulate matter chemical components and subclinical atherosclerosis: A cross-sectional analysis based on 2 advanced exposure prediction models in the multi-ethnic study of atherosclerosis. Am. J. Epidemiol..

[5] Alexeeff S.E., Schwartz J., Kloog I., Chudnovsky A., Koutrakis P., Coull B.A. (2015). Consequences of kriging and land use regression for PM_2.5_ predictions in epidemiologic analyses: Insights into spatial variability using high-resolution satellite data. J. Expo. Sci. Environ. Epidemiol..

[6] Szpiro A.A., Paciorek C.J., Sheppard L. (2011). Does more accurate exposure prediction necessarily improve health effect estimates?. Epidemiology.

[7] Szpiro A.A., Sheppard L., Lumley T. (2011). Efficient measurement error correction with spatially misaligned data. Biostatistics.

[8] Keller J.P., Chang H.H., Strickland M.J., Szpiro A.A. (2017). Measurement error correction for predicted spatiotemporal air pollution exposures. Epidemiology.

[9] Wang M., Beelen R., Bellander T., Birk M., Cesaroni G., Cirach M., Cyrys J., de Hoogh K., Declercq C., Dimakopoulou K. (2014). Performance of multi-city land use regression models for nitrogen dioxide and fine particles. Environ. Health Perspect..

[10] Allen R.W., Adar S.D., Avol E., Cohen M., Curl C.L., Larson T., Liu L.J., Sheppard L., Kaufman J.D. (2012). Modeling the residential infiltration of outdoor PM_2.5_ in the Multi-Ethnic Study of Atherosclerosis and Air Pollution (MESA Air). Environ. Health Perspect..

[11] National Reseach Council (2004). Research Priorities for Airborne Particulate Matter: IV. Continuing Research Progress.

[12] Cesaroni G., Porta D., Badaloni C., Stafoggia M., Eeftens M., Meliefste K., Forastiere F. (2012). Nitrogen dioxide levels estimated from land use regression models several years apart and association with mortality in a large cohort study. Environ. Health.

[13] Chang J.C., Hanna S.R. (2004). Air quality model performance evaluation. Meteorol. Atmos. Phys..

[14] Wang M., Beelen R., Basagana X., Becker T., Cesaroni G., de Hoogh K., Dedele A., Declercq C., Dimakopoulou K., Eeftens M. (2013). Evaluation of land use regression models for NO_2_ and particulate matter in 20 European study areas: The ESCAPE project. Environ. Sci. Technol..

[15] Wang M., Beelen R., Eeftens M., Meliefste K., Hoek G., Brunekreef B. (2012). Systematic evaluation of land use regression models for NO_2_. Environ. Sci. Technol..

[16] Zhou M., Liu Y., Wang L., Kuang X., Xu X., Kan H. (2014). Particulate air pollution and mortality in a cohort of Chinese men. Environ. Pollut..

[17] Brauer M., Amann M., Burnett R.T., Cohen A., Dentener F., Ezzati M., Henderson S.B., Krzyzanowski M., Martin R.V., van Dingenen R. (2012). Exposure assessment for estimation of the global burden of disease attributable to outdoor air pollution. Environ. Sci. Technol..

[18] Forouzanfar M.H., Alexander L., Anderson H.R., Bachman V.F., Biryukov S., Brauer M., Burnett R., Casey D., Coates M.M., GBD 2013 Risk Factors Collaborators (2015). Global, regional, and national comparative risk assessment of 79 behavioural, environmental and occupational, and metabolic risks or clusters of risks in 188 countries, 1990–2013: A systematic analysis for the Global Burden of Disease Study 2013. Lancet.

[19] Dockery D.W., Pope C.A., Xu X., Spengler J.D., Ware J.H., Fay M.E., Ferris B.G., Speizer F.E. (1993). An association between air pollution and mortality in six U.S. cities. N. Engl. J. Med..

[20] Pope C.A., Thun M.J., Namboodiri M.M., Dockery D.W., Evans J.S., Speizer F.E., Heath C.W. (1995). Particulate air pollution as a predictor of mortality in a prospective study of U.S. adults. Am. J. Respir. Crit. Care Med..

[21] Lave L.B., Seskin E.P. (1970). Air pollution and human health. Science.

[22] Chung Y., Dominici F., Wang Y., Coull B.A., Bell M.L. (2015). Associations between long-term exposure to chemical constituents of fine particulate matter (PM_2.5_) and mortality in Medicare enrollees in the eastern United States. Environ. Health Perspect..

[23] Zeger S.L., Dominici F., McDermott A., Samet J.M. (2008). Mortality in the Medicare population and chronic exposure to fine particulate air pollution in urban centers (2000–2005). Environ. Health Perspect..

[24] Chen Y., Ebenstein A., Greenstone M., Li H. (2013). Evidence on the impact of sustained exposure to air pollution on life expectancy from China’s Huai River policy. Proc. Natl. Acad. Sci. USA.

[25] Liu J., Hong Y., D’Agostino R.B., Wu Z., Wang W., Sun J., Wilson P.W., Kannel W.B., Zhao D. (2004). Predictive value for the Chinese population of the Framingham CHD risk assessment tool compared with the Chinese Multi-Provincial Cohort Study. JAMA.

[26] Wang Y., Liu J., Wang W., Wang M., Qi Y., Xie W., Li Y., Sun J., Liu J., Zhao D. (2015). Lifetime risk for cardiovascular disease in a Chinese population: The Chinese Multi-Provincial Cohort Study. Eur. J. Prev. Cardiol..

[27] Xie W., Liu J., Wang W., Wang M., Li Y., Sun J., Liu J., Qi Y., Zhao F., Zhao D. (2014). Five-year change in systolic blood pressure is independently associated with carotid atherosclerosis progression: A population-based cohort study. Hypertens. Res..

[28] Qi Y., Fan J., Liu J., Wang W., Wang M., Sun J., Liu J., Xie W., Zhao F., Li Y. (2015). Cholesterol-overloaded HDL particles are independently associated with progression of carotid atherosclerosis in a cardiovascular disease-free population: A community-based cohort study. J. Am. Coll. Cardiol..

[29] Xie W., Liu J., Wang W., Wang M., Qi Y., Zhao F., Sun J., Liu J., Li Y., Zhao D. (2016). Association between plasma PCSK9 levels and 10-year progression of carotid atherosclerosis beyond LDL-C: A cohort study. Int. J. Cardiol..

[30] Wu N., Tang X., Wu Y., Qin X., He L., Wang J., Li N., Li J., Zhang Z., Dou H. (2014). Cohort profile: The Fangshan Cohort Study of cardiovascular epidemiology in Beijing, China. J. Epidemiol..

[31] Keller J.P., Olives C., Kim S.Y., Sheppard L., Sampson P.D., Szpiro A.A., Oron A.P., Lindstrom J., Vedal S., Kaufman J.D. (2015). A unified spatiotemporal modeling approach for predicting concentrations of multiple air pollutants in the multi-ethnic study of atherosclerosis and air pollution. Environ. Health Perspect..

[32] Bergen S., Sheppard L., Sampson P.D., Kim S.Y., Richards M., Vedal S., Kaufman J.D., Szpiro A.A. (2013). A national prediction model for PM_2.5_ component exposures and measurement error-corrected health effect inference. Environ. Health Perspect..

[33] Mercer L.D., Szpiro A.A., Sheppard L., Lindstrom J., Adar S.D., Allen R.W., Avol E.L., Oron A.P., Larson T., Liu L.J. (2011). Comparing universal kriging and land-use regression for predicting concentrations of gaseous oxides of nitrogen (NO_x_) for the Multi-Ethnic Study of Atherosclerosis and Air Pollution (MESA Air). Atmos. Environ..

[34] Sampson P.D., Richards M., Szpiro A.A., Bergen S., Sheppard L., Larson T.V., Kaufman J.D. (2013). A regionalized national universal kriging model using Partial Least Squares regression for estimating annual PM_2.5_ concentrations in epidemiology. Atmos. Environ..

[35] Kaufman J.D., Adar S.D., Allen R.W., Barr R.G., Budoff M.J., Burke G.L., Casillas A.M., Cohen M.A., Curl C.L., Daviglus M.L. (2012). Prospective study of particulate air pollution exposures, subclinical atherosclerosis, and clinical cardiovascular disease: The Multi-Ethnic Study of Atherosclerosis and Air Pollution (MESA Air). Am. J. Epidemiol..

[36] Cohen M.A., Adar S.D., Allen R.W., Avol E., Curl C.L., Gould T., Hardie D., Ho A., Kinney P., Larson T.V. (2009). Approach to estimating participant pollutant exposures in the Multi-Ethnic Study of Atherosclerosis and Air Pollution (MESA Air). Environ. Sci. Technol..

[37] Vedal S., Campen M.J., McDonald J.D., Larson T.V., Sampson P.D., Sheppard L., Simpson C.D., Szpiro A.A. (2013). National Particle Component Toxicity (NPACT) Initiative Report on Cardiovascular Effects.

[38] Lindstrom J., Szpiro A.A., Sampson P.D., Oron A.P., Richards M., Larson T.V., Sheppard L. (2014). A flexible spatio-temporal model for air pollution with spatial and spatio-temporal covariates. Environ. Ecol. Stat..

[39] De Nazelle A., Seto E., Donaire-Gonzalez D., Mendez M., Matamala J., Nieuwenhuijsen M.J., Jerrett M. (2013). Improving estimates of air pollution exposure through ubiquitous sensing technologies. Environ. Pollut..

[40] Brantley H.L., Hagler G.S.W., Kimbrough E.S., Williams R.W., Mukerjee S., Neas L.M. (2014). Mobile air monitoring data-processing strategies and effects on spatial air pollution trends. Atmos. Meas. Tech..

[41] Riley E.A., Schaal L., Sasakura M., Crampton R., Gould T.R., Hartin K., Sheppard L., Larson T., Simpson C.D., Yost M.G. (2016). Correlations between short-term mobile monitoring and long-term passive sampler measurements of traffic-related air pollution. Atmos. Environ..

[42] Riley E.A., Banks L., Fintzi J., Gould T.R., Hartin K., Schaal L., Davey M., Sheppard L., Larson T., Yost M.G. (2014). Multi-pollutant mobile platform measurements of air pollutants adjacent to a major roadway. Atmos. Environ..

[43] Montagne D.R., Hoek G., Klompmaker J.O., Wang M., Meliefste K., Brunekreef B. (2015). Land use regression models for ultrafine particles and black carbon based on short-term monitoring predict past spatial variation. Environ. Sci. Technol..

[44] Hatzopoulou M., Valois M.F., Levy I., Mihele C., Lu G., Bagg S., Minet L., Brook J. (2017). Robustness of land-use regression models developed from mobile air pollutant measurements. Environ. Sci. Technol..

[45] Kerckhoffs J., Hoek G., Vlaanderen J., van Nunen E., Messier K., Brunekreef B., Gulliver J., Vermeulen R. (2017). Robustness of intra urban land-use regression models for ultrafine particles and black carbon based on mobile monitoring. Environ. Res..

[46] Wang M., Zhu T., Zheng J., Zhang R.Y., Zhang S.Q., Xie X.X., Han Y.Q., Li Y. (2009). Use of a mobile laboratory to evaluate changes in on-road air pollutants during the Beijing 2008 Summer Olympics. Atmos. Chem. Phys..

[47] Wang M., Zhu T., Zhang J.P., Zhang Q.H., Lin W.W., Li Y., Wang Z.F. (2011). Using a mobile laboratory to characterize the distribution and transport of sulfur dioxide in and around Beijing. Atmos. Chem. Phys..

[48] Jerrett M., Arain A., Kanaroglou P., Beckerman B., Potoglou D., Sahsuvaroglu T., Morrison J., Giovis C. (2005). A review and evaluation of intraurban air pollution exposure models. J. Expo. Anal. Environ. Epidemiol..

[49] Byun D., Schere K.L. (2006). Review of the governing equations, computational algorithms, and other components of the Models-3 Community Multiscale Air Quality (CMAQ) Modeling System. Appl. Mech. Rev..

[50] Simpson D., Benedictow A., Berge H., Bergström R., Emberson L.D., Fagerli H., Flechard C.R., Hayman G.D., Gauss M., Jonson J.E. (2012). The EMEP MSC-W chemical transport model—Technical description. Atmos. Chem. Phys..

[51] Zhang H., Chen G., Hu J., Chen S.H., Wiedinmyer C., Kleeman M., Ying Q. (2014). Evaluation of a seven-year air quality simulation using the Weather Research and Forecasting (WRF)/Community Multiscale Air Quality (CMAQ) models in the eastern United States. Sci. Total Environ..

[52] Wang M., Sampson P.D., Hu J., Kleeman M., Keller J.P., Olives C., Szpiro A.A., Vedal S., Kaufman J.D. (2016). Combining land-use regression and chemical transport modeling in a spatiotemporal geostatistical model for ozone and PM_2.5_. Environ. Sci. Technol..

[53] Sorek-Hamer M., Just A.C., Kloog I. (2016). Satellite remote sensing in epidemiological studies. Curr. Opin. Pediatr..

[54] Hoff R.M., Christopher S.A. (2009). Remote sensing of particulate pollution from space: Have we reached the promised land?. J. Air Waste Manag. Assoc..

[55] Lee S.J., Serre M.L., van Donkelaar A., Martin R.V., Burnett R.T., Jerrett M. (2012). Comparison of geostatistical interpolation and remote sensing techniques for estimating long-term exposure to ambient PM_2.5_ concentrations across the continental United States. Environ. Health Perspect..

[56] Friberg M.D., Zhai X., Holmes H.A., Chang H.H., Strickland M.J., Sarnat S.E., Tolbert P.E., Russell A.G., Mulholland J.A. (2016). Method for fusing observational data and chemical transport model simulations to estimate spatiotemporally resolved ambient air pollution. Environ. Sci. Technol..

[57] Lv B., Hu Y., Chang H.H., Russell A.G., Bai Y. (2016). Improving the accuracy of daily PM_2.5_ distributions derived from the fusion of ground-level measurements with aerosol optical depth observations, a case study in North China. Environ. Sci. Technol..

[58] Lee M., Kloog I., Chudnovsky A., Lyapustin A., Wang Y., Melly S., Coull B., Koutrakis P., Schwartz J. (2016). Spatiotemporal prediction of fine particulate matter using high-resolution satellite images in the Southeastern U.S. 2003–2011. J. Expo. Sci. Environ. Epidemiol..

[59] Kloog I., Nordio F., Coull B.A., Schwartz J. (2012). Incorporating local land use regression and satellite aerosol optical depth in a hybrid model of spatiotemporal PM_2.5_ exposures in the Mid-Atlantic states. Environ. Sci. Technol..

[60] Wilton D., Szpiro A., Gould T., Larson T. (2010). Improving spatial concentration estimates for nitrogen oxides using a hybrid meteorological dispersion/land use regression model in Los Angeles, CA and Seattle, WA. Sci. Total Environ..

[61] Young M.T., Bechle M.J., Sampson P.D., Szpiro A.A., Marshall J.D., Sheppard L., Kaufman J.D. (2016). Satellite-Based NO_2_ and model validation in a national prediction model based on universal kriging and land-use regression. Environ. Sci. Technol..

[62] Chi G.C., Hajat A., Bird C.E., Cullen M.R., Griffin B.A., Miller K.A., Shih R.A., Stefanick M.L., Vedal S., Whitsel E.A. (2016). Individual and neighborhood socioeconomic status and the association between air pollution and cardiovascular disease. Environ. Health Perspect..

